# Supplementary material for: Growth, Safety and Tolerance in Infants Fed Rice Protein Hydrolysate Formula: The GRITO Randomised Controlled Trial
Source: Nutrients. 2024 Dec 31;17(1):162. doi: 10.3390/nu17010162 (PMC11722687; doi:10.3390/nu17010162)

Supplementary Material

Supplementary Table S1. Anthropometric data by study visit

		Weight (g)		Length (cm)		BMI (Kg/m^2)		Head circumfer-ence (cm)		Triceps skin fold (mm)		Mid-arm circumference (cm)		Arm muscle area (cm^2)	
		**eHF**	**HRF**	**eHF**	**HRF**	**eHF**	**HRF**	**eHF**	**HRF**	**eHF**	**HRF**	**eHF**	**HRF**	**eHF**	**HRF**
**BASELINE**	N	48	44	48	44	48	44	48	45	48	45	48	44	47	44
	Mean	7.406	7.686	66.90	67.23	16.44	16.92	43.48	43.36	9.92	10.11	13.33	13.80	10.26	10.64
	SD	1.314	1.274	5.56	4.51	1.19	1.54	3.26	2.38	2.66	2.55	1.79	1.90	3.72	3.58
**1 MONTH**	N	44	42	44	42	44	42	44	43	44	43	43	43	43	43
	Mean	7.847	8.149	68.20	68.57	16.83	17.26	43.66	44.30	10.45	10.44	13.86	14.56	11.33	11.53
	SD	1.131	1187	5.02	4.10	1.31	1.45	2.60	2.58	2.45	2.37	2.13	1.75	4.30	4.45
**2 MONTHS**	N	48	42	48	42	48	42	48	42	48	42	48	42	46	42
	Mean	8.329	8.344	70.19	69.86	16.89	17.05	44.73	44.81	9.83	10.24	14.19	14.81	11.70	12.02
	SD	1.119	1.158	4.91	3.80	1.41	1.60	2.44	2.23	2.39	3.01	1.50	1.63	4.29	4.51
**4 MONTHS**	N	43	43	43	43	43	43	43	43	43	43	43	43	42	43
	Mean	9.015	9.103	73.02	72.72	16.90	17.17	45.56	45.93	10.23	10.23	14.33	14.81	11.83	11.98
	SD	1.137	1.201	4.77	3.59	1.45	1.56	2.06	2.37	2.28	2.62	1.77	1.62	3.93	4.50
**6 MONTHS**	N	42	41	43	41	42	41	41	41	41	41	41	41	40	41
	Mean	9.455	9.571	75.23	74.98	16.76	16.99	46.02	46.54	10.05	9.68	14.68	14.78	11.63	12.63
	SD	1.068	1.147	4.06	3.52	1.50	1.36	1.84	2.19	2.54	3.07	1.63	1.35	3.73	4.49
**9 MONTHS**	N	28	19	28	19	28	19	27	19	27	19	27	19	27	19
	Mean	10.106	10282	77.61	77.84	16.78	16.91	46.41	46.68	9.85	9.32	14.74	14.95	12.11	12.89
	SD	1.026	1374	3.44	4.63	1.34	1.04	1.82	2.29	2.55	2.75	1.81	1.39	4.25	3.83
**12 MONTHS**	N	19	18	19	18	19	18	17	18	19	18	18	18	18	18
	Mean	10.828	10600	81.32	80.17	16.22	16.47	47.24	47.28	10.95	8.83	15.28	15.06	13.89	13.78
	SD	1391	1376	3.02	4.23	1.27	1.55	1.89	1.64	2.50	2.98	2.85	1.39	5.36	4.86

Supplementary Table S2. Digestive tolerance during follow-up

	eHF	HRF	*p*
Mean stool frequency (stools/day)			
6 months	2.89	2.75	0.80
9 months	3.31	3.39	0.65
12 months	2.67	2.98	0.55
Mean daily number of regurgitations			Group effect: $p = 0.506$
6 months	0.42	0.05	Time effect: $p < 0.001$
9 months	0.28	0.17	
12 months	0.10	0.038	
Odds Ratio [95%CI] at 6 months	1 (Reference)	0.18 [0.33-0.95]	0.04
Odds Ratio [95%CI] at 9 months	1 (Reference)	0.38 [0.08-1.38]	0.21
Odds Ratio [95%CI] at 12 months	1 (Reference)	0.31 [0.03-3.19]	0.33
Mean daily number of colic episodes			Group effect: $p = 0.965$
6 months	0.23	0.12	Time effect: 0.0450
9 months	0.13	0.24	
12 months	0.07	0.18	
Odds Ratio [95%CI] at 6 months	1 (Reference)	0.38 [0.07-1.90]	0.24
Odds Ratio [95%CI] at 9 months	1 (Reference)	1.41 [0.28-7.16]	0.68
Odds Ratio [95%CI] at 12 months	1 (Reference)	0.71 [0.09-5.44]	0.74

Supplementary Table S3. Severe adverse events

AE	Intensity	Relation	Actions	Outcome	Group
Not described	Moderate	Unrelate	Hospitalisation	Resolved	eHF
Pneumonia	Moderate	Unrelate	Hospitalisation	Resolved	eHF
Pneumonia	Moderate	Unrelate	Hospitalisation	Resolved	HRF
Bronchiolitis	Moderate	Unrelate	Hospitalisation	Resolved	eHF
Bronchiolitis	Moderate	Unrelate	Hospitalisation	Resolved	HRF
Bronchospasm	Moderate	Unrelate	Hospitalisation	Resolved	HRF
Bronchospasm	Moderate	Unrelate	Hospitalisation	Resolved	HRF
Bronchospasm	Moderate	Unrelate	Hospitalisation	Resolved	eHF
Gastroenteritis[1]	Mild	Unrelate	Medication	Resolved	eHF
Gastroenteritis	Mild	Unrelate	Hospitalisation	Resolved	eHF
Anaphylactic reaction[2]	Moderate	Unrelate	Hospitalisation	Resolved	HRF
Decrease of the quantity of randomised formula from 900ml to 200ml[3]	Severe	Unrelate	Hospitalisation	Resolved	eHF
Disordered eating behaviour	Mild	Unrelate	Hospitalisation	Resolved	eHF
Disordered eating behaviour	Mild	Unrelate	Hospitalisation	Resolved	eHF
Disordered eating behaviour	Moderate	Unrelate	Hospitalisation	Resolved	eHF
Weight stagnation	Moderate	Unrelate	Hospitalisation	Resolved	eHF

1- vital prognosis threatened;
2- cause not documented, but unrelated to the study product.
3- Infant with medical history of gastroesophageal reflux /esophagitis. The reported event was anorexia.

Supplementary Table S4. Adverse events related to the formula

	HRF	eHF	TOTAL
**Number of patients with AEs related to the formula**	**6**	**9**	**15**
**Number of AEs**	**12**	**17**	**29**
**Causality**			
Certainly related to the product	1	1	2
Probably related to the product	11	7	18
Unlikely to be related to the product	0	9	9
**Severity of AE**			
Mild	9	10	19
Moderate	3	7	10

Supplementary Table S5. Details of adverse events by formula

Related AEs	HRF	eHF	TOTAL
Allergic reaction to cow's milk	0	1*	1
Constipation	5	2°	7
Abdominal pain	4	0	4
Regurgitation	0	4	4
Atopic dermatitis	0	2*	2
Bronchiolitis	0	1*	1
Cutaneous eruption	1	0	1
Diarrhoea	0	1*	1
Diarrhoea and vomiting	1	0	1
Facial oedema	0	1	1
Gastroenteritis	0	1*	1
Malaise	0	1*	1
Otitis	0	1*	1
Rectal bleeding	1	0	1
Urticaria	0	1	1
Vomiting	0	1	1
**TOTAL**	**12**	**17**	**29**

** Unlikely to be related to the product*

° one case of constipation as considered unlikely to be related to the product

Supplementary Figure S1. Study design.

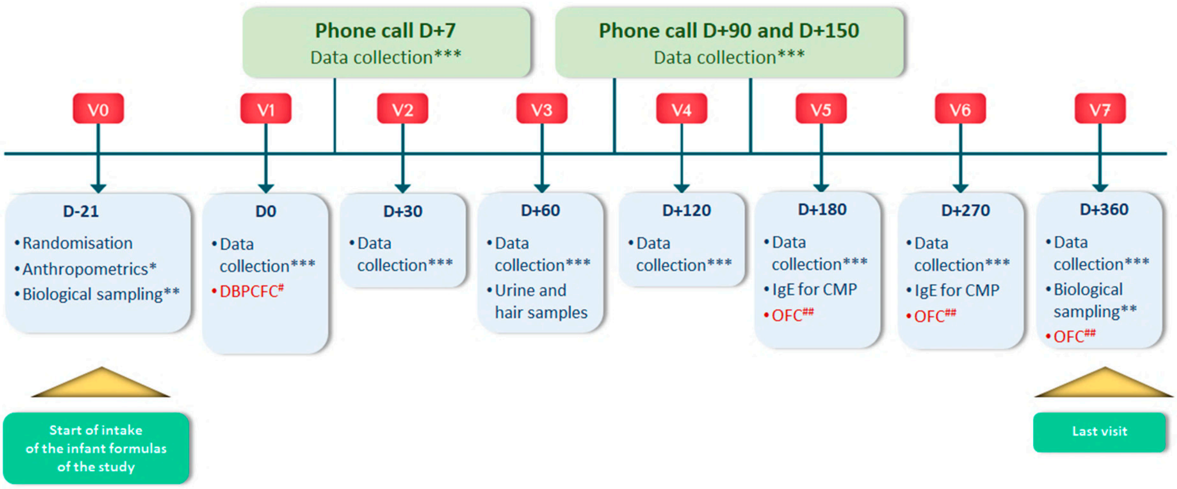

***Anthropometrics**: weight, length, weight/ length, body mass index (BMI), head circumference, triceps skin fold, mid arm circumference, arm muscle area
****Biological sampling:** blood samples notably specific IgE for CMPA (against alpha-lactalbumin, beta-lactoglobulin, caseins or whole milk); Urine and hair samples to notably determine arsenic levels.
*****DATA collection**: concomitant medication, clinical evaluation, anthropometrics, adverse events
#**DBPCFC**: Double-Blind Placebo-Control Food Challenge
##**OFC:** Open Oral Food Challenge. Patients with a negative oral food challenge (no reaction) were shifted to a standard milk formula and were followed until the end of the study to both primary and secondary endpoints anthropometric parameters.

Supplementary Figure S2. Patient flow diagram.

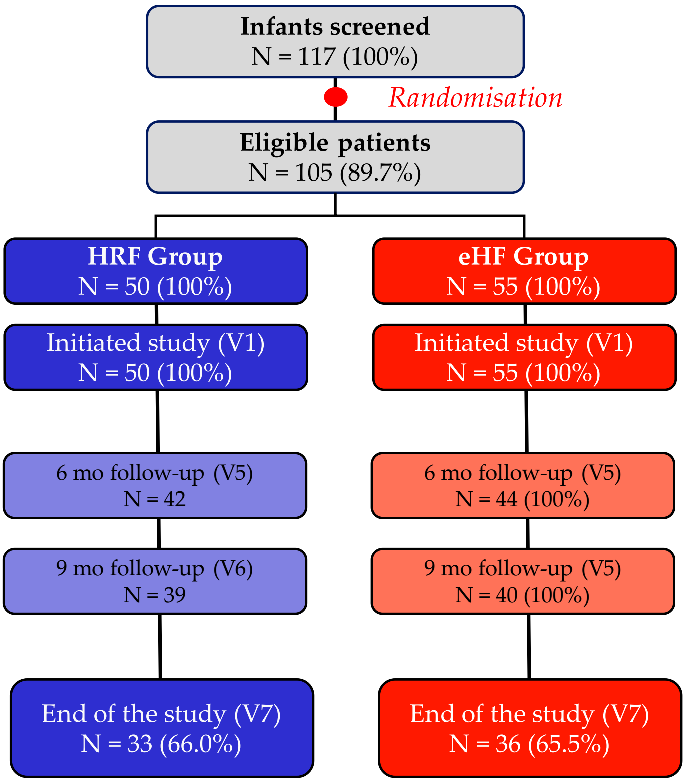

Supplement: Supplementary file 1 [file nutrients-17-00162-s001.zip › nutrients-3412465-supplementary.pdf]
